# Alterations of Gut Microbiota and the Brain-Immune-Intestine Axis in Patients With Relapsing-Remitting Multiple Sclerosis After Treatment With Oral Cladribine: Protocol for a Prospective Observational Study

**DOI:** 10.2196/16162

**Published:** 2020-07-29

**Authors:** Jeske van Pamelen, Lynn van Olst, Andries E Budding, BIA Study Group, Helga E de Vries, Leo H Visser

**Affiliations:** 1 Department of Neurology Elisabeth-TweeSteden Ziekenhuis Hospital Tilburg Netherlands; 2 Department of Molecular Cell Biology and Immunology MS Center Amsterdam Amsterdam University Medical Centers Amsterdam Netherlands; 3 inBiome Amsterdam Netherlands; 4 see Acknowledgments; 5 Department of Medical Biochemistry Amsterdam Cardiovascular Sciences University of Amsterdam Amsterdam Netherlands

**Keywords:** multiple sclerosis, cladribine, brain-immune-intestine axis, brain-gut axis, gut-brain axis, microbiota

## Abstract

**Background:**

Immunological factors are the key to the pathogenesis of multiple sclerosis (MS). Conjointly, environmental factors are known to affect MS disease onset and progression. Several studies have found that the intestinal microbiota in MS patients differs from that of control subjects. One study found a trend toward lower species richness in patients with active disease versus in patients in remission. The microbiota plays an important role in shaping the immune system. Recent studies suggest the presence of an association between the gut microbiota and inflammatory pathways in the central nervous system. However, the function of this brain-immune-intestine axis and its possible value for predicting treatment effect in MS patients is currently unknown.

**Objective:**

Our goal is to examine if the changes in gut and oral microbiota and simultaneous changes in the immune response are a predictor for the treatment response in subjects with active relapsing-remitting MS (RRMS) who are being treated with oral cladribine.

**Methods:**

This is a prospective, observational, multicenter study. Eligible subjects are patients with RRMS, between the ages of 18 and 55 years, who will start treatment with oral cladribine. Patients who used probiotics 1 month prior to the start of oral cladribine will be excluded. At baseline (ie, before start) and after 3, 12, and 24 months, the Expanded Disability Status Scale (EDSS) score will be assessed and fecal, oral, and blood samples will be collected. Also, subjects will be asked to register their food intake for 7 consecutive days following the visits. After 24 months, a magnetic resonance imaging (MRI) assessment of the brain will be performed. Responders are defined as subjects without relapses, without progression on the EDSS, and without radiological progression on MRI.

**Results:**

Inclusion started in January 2019. A total of 30 patients are included at the moment. The aim is to include 80 patients from 10 participating centers during a period of approximately 24 months. Final results are expected in 2024.

**Conclusions:**

The results of the BIA Study will contribute to precision medicine in patients with RRMS and will contribute to a better understanding of the brain-immune-intestine axis.

**International Registered Report Identifier (IRRID):**

DERR1-10.2196/16162

## Introduction

Immunological and environmental factors are known to be important in the pathogenesis of multiple sclerosis (MS) [1-3]. Recent studies have found that the gut microbiota in MS patients is different from healthy controls [4-7]. One study found a trend toward a decreased species richness in patients with active disease compared to healthy controls and patients who are in remission [6]. The microbiota plays an important role in shaping the immune system, and recent studies suggest an association between the gut and the central nervous system inflammatory demyelination [2-4,7-11].

The connection between microbiota, treatment, and changes in immunity in MS has not yet been examined well. Several recent studies showed that MS patients treated with interferon, glatiramer acetate, or dimethyl fumarate had an altered microbiota in comparison to the patients who did not use immunomodulatory treatment (IMT) [4,8,12].

For cladribine, it is known that after 2 years approximately 50% of the patients have reached the goal of no evidence of disease activity-3 (NEDA-3); that is, patients do not have relapses, show no disability progression, and do not have active disease as seen by magnetic resonance imaging (MRI), as measured by the number and volume of the T2 lesions and the presence of gadolinium-enhancing lesions on T1 images [13]. Cladribine is also used for the treatment of celiac disease, a condition in which there is inflammation of the small intestine due to exposure to gluten. It has been shown that cladribine has a positive effect on the gut immunological system [14].

This study aims to investigate whether the gut and oral microbiota, or changes in gut and oral microbiota after therapy, are a predictor for treatment response in subjects with active relapsing-remitting MS (RRMS) and whether these changes result in downgrading the immune response. To our knowledge, this is the first study to investigate changes in microbiota and immune response in patients with RRMS in a longitudinal follow-up setting.

## Methods

### Study Objectives and Hypotheses

The main aim of this study is to determine if the gut and oral microbiota at baseline, or the change in gut and oral microbiota in the first 3 months after start of cladribine, is a predictor for treatment response in subjects with active RRMS. The time point of 3 months after the start of treatment was chosen in order to be able to predict early in the treatment regimen whether a treatment response can be expected. Only one previous study investigated changes in microbiota after IMT and found a trend toward changes in microbiota after 2 and 12 weeks [15]. However, possibly due to a small sample size, after correction for multiple comparisons, none of their results seem to be significant. Nevertheless, we expect that 12 weeks (ie, 3 months) is a good moment to measure changes, because changes in different immune cell subsets after administration of oral cladribine have been shown to already appear after 5 weeks and stabilize after 13 weeks [16]. The hypothesis that there is an interaction between the immune system and gut microbiota suggests that we can also expect changes in microbiota at that moment.

The secondary objectives are to answer the following questions:

1. What is the difference between the gut and oral microbiota in patients who experience disease activity (ie, relapse, radiological activity, or disability progression) in comparison to patients without disease activity at baseline and at 3, 12, or 24 months?
2. What is the difference between the immune profile in patients who experience disease activity in comparison to patients without disease activity at baseline and at 3, 12, or 24 months?
3. Will cladribine treatment result in a more balanced microbial profile and a less inflammatory immune profile at 3, 12, or 24 months?

Our hypothesis is that RRMS patients treated with oral cladribine who have NEDA-3 after a follow-up period of 2 years will have a more balanced microbial profile together with a reduced proinflammatory profile of circulating immune cells when compared to the patients who do not have NEDA-3 after 2 years of follow-up. We expect that this study may contribute to a better understanding of the association between the gut, the immune system, and the central nervous system in patients with RRMS.

### Design

The BIA (brain-immune-intestine axis) Study is a prospective, observational, multicenter study. Subjects with active RRMS who start with oral cladribine treatment, as per standard of care, will be asked to participate in this study. Participation includes four to five visits over 24 months.

### Population

Patients will be recruited from the outpatient clinics of the participating neurological departments. Enrollment will take place at 10 participating centers in the Netherlands during a period of approximately 24 months. Eligible subjects are patients with clinically definite RRMS, between the ages of 18 and 55 years, with a planned start of cladribine treatment. We excluded patients who used probiotics within 1 month prior to the planned start of cladribine. We have chosen not to exclude patients with other potentially confounding factors, such as recent treatment with antibiotics or corticosteroids and a medical history of bowel disease, to make sure our population reflects daily practice. However, these factors will be collected throughout the study to be able to take them into account during analysis.

### Sample Size Calculation

This is an exploratory study. No previous studies assessing the primary outcome have been performed. Other studies examining the gut microbiota in MS patients usually included 30-60 patients [4,6-8,11]. With 30-60 patients, these studies were able to clearly show differences in the gut microbiota between MS patients and healthy controls. We want to assess whether there is a difference in the gut microbiota in responders versus nonresponders in patients treated with oral cladribine. Previous studies with oral cladribine in MS patients have shown that approximately half of the patients will reach NEDA-3.

In order to obtain sufficient statistical power for a reliable prediction, we follow the rule of thumb of having at least 10 patients per event [17].

Taking into account that about 50% of the participants will have the event (ie, disease activity, which can include relapses, disability progression, or radiological progression) and that four facets of the gut microbiota profile will be used to predict the event, we need 80 patients (ie, 4 × 10 × 2 = 80). These four facets will be derived from the different approaches described in the Statistical Analysis section of this protocol.

### Ethics Committee Approval

This study was approved by the medical ethics committee of Brabant, based in Tilburg, the Netherlands. Written informed consent will be obtained from all participants.

### Procedures

This study will include four to five visits—a screening and baseline visit and visits after 3, 12, and 24 months—of which only the baseline visit is an additional visit. Procedures in the screening visit can be partly performed during the baseline visit or by telephone contact. All other visits will be combined with routine hospital visits. During these visits, patients will undergo neurological examination and several study-specific invasive and noninvasive measurements. Blood samples and an oral swab will be taken at baseline and after 3, 12, and 24 months, and one additional MRI (ie, T1-weighted, T2-weighted, fluid-attenuated inversion recovery [FLAIR], and T1-weighted imaging with gadolinium) will be performed after 2 years. A stool sample will be collected at baseline and at months 3, 12, and 24, and subjects will be asked to rate their feces on the Bristol Stool Scale. Subjects will also be asked to register their food and drink intake for 7 consecutive days following the baseline visit and the visits at months 3, 12, and 24.

Table 1 provides an overview of all procedures per visit. A distinction is made between routine assessments and study-specific assessments.

The treating physician will be responsible for all procedures, which can be partially delegated to a research nurse. MRIs will be assessed by a central reader. A central laboratory will analyze gut and oral microbiota from stool samples and oral swabs and an immunological profile from blood samples.

**Table 1:** Schedule of visits and assessments.

Assessment	Screening visit: Day –28 to 0^a^	Baseline visit: Day –7 to 0^a^	Visit at 3 months	Visit at 12 months	Visit at 24 months	Unscheduled visit (<10 days of relapse)
Informed consent	X^b^					
Inclusion and exclusion criteria check	X	X				
Demography	Y^c^					
Medical history	Y					
Height	Y					
Weight	Y			Y		
Vital signs	Y	X	Y	Y	Y	
Bristol Stool Scale		X	X	X	X	
Disease assessment: Expanded Disability Status Scale (EDSS)	X		X	X	X	X
Magnetic resonance imaging (MRI): brain	Y			Y	X	
Oral swab		X	X	X	X	
Stool sample collection for microbiota assessment		X	X	X	X	
Blood sampling for immunological assessment		X	X	X	X	
Concomitant medication	Y		Y	Y	Y	Y
Adverse events		Y	Y	Y	Y	Y
Food registration by patient		X	X	X	X	

^a^Screening and baseline visit activities can be performed on the same day.

^b^X: study-specific assessment.

^c^Y: routine assessment.

### Withdrawal of Subjects

Subjects can leave the study at any time for any reason if they wish to do so without any consequences. The investigator can decide to withdraw a subject from the study for urgent medical reasons. Subjects who discontinue cladribine, prior to completing the week 2 intake in the second year, and switch to another MS medication will be asked to continue participation in the BIA Study. The new MS medication will be documented as concomitant medication.

### Materials and Measures

#### Expanded Disability Status Score

During the screening visit and the visits at months 3, 12, and 24, the Expanded Disability Status Scale (EDSS) score will be recorded by performing a neurological examination. In case the subject experiences a relapse between screening and baseline, the EDSS will have to be repeated at the Day 0 visit to assess a new baseline value.

#### Microbiota Assessment

Oral swabs and stool samples will be collected using eNAT tubes (Copan Diagnostics, Inc) at baseline and at months 3, 12, and 24 for microbiota assessment. These tubes contain a guanidine-thiocyanate-based medium that stabilizes RNA and DNA and can be stored at room temperature for 4 weeks and at –20°C after that [18]. This allows patients to collect their stool samples at home and bring them to their hospital visits. Oral swabs will be collected during their hospital visits. After the visits, both tubes will be stored at –20°C until analysis. Composition of the gut microbiota will be determined using the IS-pro (interspace profiling) technique, a clinically validated molecular assay for analysis of complex microbiota [19]. The IS-pro technique (inBiome) identifies bacteria based on specific-length polymorphisms in the 16S-23S rDNA interspace (IS) region, combined with phylum-specific sequence polymorphisms in the 16S rDNA. Resulting data consist of peak profiles, with different colors relating to the different phylum groups (ie, Bacteroidetes; Proteobacteria; and Firmicutes, Actinobacteria, Fusobacteria, and Verrucomicrobia) and length signatures corresponding to the specific species.

#### Blood Samples

Blood samples will be drawn at baseline and at months 3, 12, and 24 for immunological assessment. Blood will be collected in cell preparation tubes, which allow for direct isolation of the peripheral blood mononuclear cells (PBMCs). Cells will be stored at –80°C for a maximum of 3 months and then in liquid nitrogen. Subsequently, the cells will be used for single-cell mass cytometry analysis using cytometry by time-of-flight (CyTOF).

#### Bristol Stool Scale

Subjects will be asked to rate their feces on the Bristol Stool Scale at baseline and at months 3, 12, and 24. This information will be used to correct for stool consistency. Previous research investigating features that accounted for gut microbiome variation showed that the Bristol Stool Scale score is the most important feature covarying with fecal microbiome composition [20].

#### Food Intake Registration

Subjects will be requested to record their food intake for 7 consecutive days following the baseline visit and visits at 3, 12, and 24 months. This information will be used to correct for diet as a potential confounder (eg, vegetarian diet). Also, we will use this information to check for changes in diet throughout the study period, which could possibly explain some changes in the microbiota.

#### MRI

Gadolinium-enhanced MRI of the brain will be performed at the screening visit, after 12 months (ie, standard care) and after 24 months (ie, study-specific assessment). This MRI will include FLAIR, T2-weighted, and T1-weighted scanning before and after intravenous gadolinium has been given. The MRI parameters will be as follows: T1-lesion load, T2-lesion load, number of enhancing lesions, and number of new and enlarging lesions. If a routine (ie, standard care) MRI has been made within 3 months prior to the start of cladribine, this MRI will not have to be repeated.

#### Other Measures

At the screening visit, demographic data; medical history, including MS disease activity and gastrointestinal diseases; prior use of IMTs, antibiotics, and glucocorticoids; height and weight; and smoking status will be recorded. At month 12, weight will again be recorded. Vital signs, including blood pressure and pulse, will be measured at screening and baseline visits and at months 3, 12, and 24 visits. Concomitant medication and adverse events, both serious and nonserious, will be registered throughout the study. All these measures can be used to find out whether they are important confounders for microbiota composition.

### Statistical Analysis

#### Primary Study Parameters

The primary study parameter is difference in gut and oral microbiota at baseline or during the first 3 months after the start of oral cladribine between responders and nonresponders. Response to cladribine treatment will be determined using various data:

1. EDSS: score ranging from 0.0 (normal neurological examination) to 10.0 (death due to MS).
2. MRI (brain): radiological changes, including T1-lesion load, T2-lesion load, number of enhancing lesions, and number of new and enlarging lesions, as assessed by a central reader.
3. Documented relapses.

We will evaluate whether responders and nonresponders have a distinct gut and oral microbiota and whether they can be classified based on intestinal or oral microbiota composition prior to therapy and after an initial period of 3 months after the first treatment.

Microbiota composition will be evaluated in different ways. As microbiota data contain many variables, a first approach is to reduce the number of variables. The most commonly used approaches, that we will also use here, are to calculate the diversity of the microbiota with the Shannon diversity index and to analyze the microbiota on the phylum level, a high-level taxonomic classification. Differences between responders and nonresponders in Shannon diversity indices and phylum abundance will be calculated by classical statistical approaches, the Mann-Whitney U test, or the Student *t* test where appropriate. Bonferroni corrections will be applied where appropriate.

A second approach will be evaluation by time series analysis. Using these longitudinal analyses, subjects will act as their own control. An important advantage of this type of analysis is that the impact of confounding factors is small, because samples are derived from one subject. Changes in potentially confounding factors, such as diet or recent use of antibiotics, can be accounted for based on knowledge from previous studies [21-23]. Changes found using this analysis can be used to create insight into potentially useful parameters to predict response to treatment and can be used in the following approaches.

A third approach for evaluation will be by unsupervised clustering of microbiota profiles. This will be done by generating a correlation matrix based on cosine-correlations of paired microbiota profiles. The matrix will be further clustered with the unweighted-pair group method with arithmetic mean. Resulting clusters will be analyzed for over- or underrepresentation of responders or nonresponders, again using the Mann-Whitney U test or the Student *t* test where appropriate.

The fourth approach for evaluation that we will use is a supervised classification approach. The classifier of choice is adaptive group-regularized logistic ridge regression (AGRR). This classifier has several advantages. First, it enables estimation and predictor selection when the number of features (ie, bacterial features) exceeds the number of observations. Hence, in contrast to standard classifiers, it can deal with high-dimensional data. Second, it allows for the structural use of codata in order to improve predictive performance. Codata refers to additional information on the measured variables. In this case, we will have information on the phylum that each bacterial feature belongs to. Considering this information implies that we will take into account that phylum composition may have additional predictive value. Moreover, information on predictive power at the phylum level will also facilitate feature selection (eg, if Bacteroidetes are most predictive, then the model will give more weight to the selection of bacteria belonging to this phylum). The prediction model will include corrections for clinical variables, such as gender and age, and will take into account potential confounders. Because of a relatively small sample size, we will consider which are the most important confounders based on previous findings and use stratification of these confounders into only a small number of categories, to make sure there are enough subjects in each category. The AGRR depends, as do all regularized classifiers, on penalty parameters. Tuning of these penalties will rest on efficient cross-validation and empirical Bayes estimation. Predictive performance of the model will be assessed by receiver operating characteristic (ROC) curves and area under the ROC curves based on cross-validated predictions obtained from 10-fold cross-validation. The AGRR was developed and implemented by the Statistics for Omics group of the Department of Epidemiology and Biostatistics of the Vrije Universiteit Medical Center [24].

Using these four approaches, we can evaluate our data and decide which parameters can be used to create a model that can predict whether a patient will or will not respond to therapy.

#### Secondary Study Parameters

Differences between responders and nonresponders in the composition of the gut and oral microbiota after 12 and 24 months will be determined using the same test as used for baseline samples and samples after 3 months.

High-dimensional immune profiling of the RRMS patients on cladribine will be performed using the Maxpar Direct Immune Profiling System (Fluidigm) with CyTOF. This assay uses a 30-marker antibody panel and automated Maxpar Pathsetter software (Fluidigm) that can identify 37 immune cell populations based on the single-cell expression of 30 protein markers. The software is developed using probability state modelling, eliminates the variability of manual gating, and provides an efficient and reliable solution for data analysis in longitudinal and multisite studies.

For each sample, 500,000 PBMCs will be measured on a single-cell level using a Helios mass cytometer (Fluidigm). Subsequently, CyTOF Software v6.7 for Maxpar Direct Immune Profiling Assay (v6.7.1016 or higher) with default flow cytometry software (FCS) processing settings will be used to normalize the final FCS files. After sample acquisition and data normalization, normalized FCS files will be analyzed using the above-mentioned Maxpar Pathsetter software.

## Results

Participant inclusion started in January 2019. A total of 30 patients are included at the moment. The aim is to include 80 patients from 10 participating centers during a period of approximately 24 months. Final results are expected in 2024.

## Discussion

The BIA Study investigates whether patients with active RRMS on oral cladribine, with or without disease activity after 2 years, have a distinct gut and oral microbiota and an altered immune profile. The results may be applicable in daily practice when deciding which patient is likely to have a response to oral cladribine or, after 3 months, which patient should switch to another immunomodulatory drug. Unnecessary side effects, potential risks, and useless treatment can be avoided this way. Also, this study may contribute to a better understanding of the association between the gut and central nervous system inflammation.

This study has several strengths. First, to our knowledge, this is the first study to investigate changes in gut microbiota and immune response in a longitudinal follow-up setting in patients with RRMS on oral cladribine treatment. Prior studies have only investigated gut microbiota profiles and immune responses in a cross-sectional design, which means they compared samples of different subgroups with each other or compared samples of patients with samples of healthy controls [4-8,11,12]. Changes due to IMT can be measured only on a group level this way. Interindividual variability in microbiota profiles is high, so it is better to let each subject act as his or her own control. In this study, due to its longitudinal design, we will be able to describe the changes in microbiota and the immune response due to cladribine on an individual level. Also, we will be able to determine the predictive value of the changes in the microbiota and/or immune system on the treatment effect.

Second, this is the first clinical study to combine investigation of changes in the gut microbiota with the oral microbiota. If changes in oral microbiota can be found that are equally predictive as those found in the gut microbiota, this would be highly beneficial for clinical implementation, as oral samples are much easier to obtain than fecal samples.

Third, this is the first study to investigate the effect of cladribine on the immune system on a high-dimensional and single-cell level using CyTOF. These data will not only give extensive insight into the effect of cladribine on the immune system, but also on the role the immune system plays in responders versus nonresponders.

This study also has several limitations. First, subjects were allowed to use IMT prior to the start of oral cladribine. The baseline samples can, therefore, be influenced by this therapy. Also, we did not exclude other confounding factors, such as recently used antibiotics or corticosteroids and bowel disease. However, we will collect data on all these potential confounders in order to take them into account. Also, we will collect data on the consistency of the feces and on dietary habits, which are known confounders [1,20].

Second, generalizability of results may be limited because subjects only used oral cladribine and no other IMT. However, previous research has shown that different IMTs influence the same inflammatory pathways, so it may provide an excellent basis for further research on the brain-immune-intestine axis [12]. Cladribine was chosen for several reasons: its approximately 50% response rate, to create even groups; its known positive effect on the gut immunological system from previous research in celiac disease; and to include a more homogeneous group of patients, based on type of MS, disease severity, and type of IMT [13,25].

In summary, the results of the BIA Study are likely to contribute to more individualized medicine in patients with RRMS and to contribute to a better understanding of the brain-immune-intestine axis.

## Abbreviations

AGRR: adaptive group-regularized logistic ridge regression
BIA: brain-immune-intestine axis
CyTOF: cytometry by time-of-flight
EDSS: Expanded Disability Status Scale
FCS: flow cytometry software
FLAIR: fluid-attenuated inversion recovery
IMT: immunomodulatory treatment
IS: interspace
IS-pro: interspace profiling
MRI: magnetic resonance imaging
MS: multiple sclerosis
NEDA: no evidence of disease activity
PBMC: peripheral blood mononuclear cell
ROC: receiver operating characteristic
RRMS: relapsing-remitting multiple sclerosis
